# Miltefosine: A Miracle Drug for Meningoencephalitis Caused by Free-Living Amoebas

**DOI:** 10.7759/cureus.13698

**Published:** 2021-03-04

**Authors:** Ammar Alli, Juan Fernando Ortiz, Álvaro Morillo Cox, Maria Armas, Victor A Orellana

**Affiliations:** 1 Internal Medicine, Tishreen University Faculty of Medicine, Lattakia, SYR; 2 Internal Medicine, Universitat de Barcelona, Barcelona, ESP; 3 Neurology, Universidad San Francisco de Quito, Quito, ECU; 4 Neurology, Larkin Community Hospital, Miami, USA; 5 Medicine, Universidad San Francisco de Quito, Quito, ECU; 6 Surgery, Pontificia Universidad Catolica del Ecuador, Quito, ECU; 7 Obstetrics and Gynecology, Pontifica Universidad Católica del Ecuador, Quito, ECU

**Keywords:** free living amoebas, meningoencephalitis

## Abstract

Meningoencephalitis caused by free-living amoebas (FLA) has a high mortality rate, and most treatments are ineffective. FLA includes Naegleria, Fowleri, Acanthamoeba, and Balamuthia mandrillaris (M). We explore the use of miltefosine in the treatment of one of these infections. The concerning mortality of the infection obligates us to look for more effective treatments for meningoencephalitis caused by FLA. During this review, we will consolidate the knowledge of using miltefosine in these three infections. We will investigate the mechanism by which the drug is effective in these infections as well. After this comprehensive review, we should assess if miltefosine improves the mortality and prognosis of the infection with the information collected. We used a Medical Subject Headings (MeSH) search on PubMed. Inclusion criteria included papers written in the English language and human subjects research for the past 25 years. Until today, there are no definitive guidelines to be followed when treating such patients. However, miltefosine has demonstrated promising results. Miltefosine decreases the usual mortality rate in the three infections; however, there are few reports due to the low frequency of these infections. Almost all cases we documented have survived. More information needs to be gathered for the use of miltefosine for these infections.

## Introduction and background

Naegleria causes primary amebic meningoencephalitis (PAM). The parasite lives in temperatures above 30 degrees and can tolerate temperatures up to 45 degrees. The most common risk factor in developing the infection is participating in recreational water activities. Approximately 300 cases have been reported worldwide [1]. Inhalation of infected water leads to the involvement of the olfactory bulb. Extra central nervous system (CNS) infection also has been reported [1]. In a CDC report, 79% of cases were male, and infections occurred mainly in the July-September period in the southern states [2]. For PAM, the median time from exposure to death is 9.9 days [3]. In a report of 111 cases, mortality was 99% [3]. The main symptoms of the disease are neurological. In the end, the disease presents rapid deterioration with profound mental alteration and severe intracranial hypertension leading to herniation and death a few days after the onset of symptoms [4]. The diagnosis begins by having a high level of suspicion related to the exposure and the patient's rapid clinical deterioration. The key is to perform a lumbar puncture with cerebrospinal fluid (CSF) analysis by a phase-contrast microscope is beneficial to ameba visualization. Giemsa or trichrome stains help define morphology features [5].

Acanthamoeba causes granulomatous amebic encephalitis (GAE), mainly among immunocompromised patients; however, immunocompetent cases have also been reported [6]. The transmission mode includes the inhalation of the cyst through the respiratory tract or direct contact with the skin followed by hematogenous spread. The parasite is usually located in environments with rich biofilms like sewage, heating hospital environments, dental and dialysis units, and contact lenses [7]. Approximately 150 cases have been reported worldwide [8]. The median time from exposure to death is eight days to a few months and the mortality rate ranges from 97% to 98% [6]. The clinical signs and symptoms of GAE are headaches, neck stiffness, confusion, gait ataxia, irritability, hemiparesis, diplopia, photophobia, and cranial nerve palsies [9]. Clinical suspicion and history are needed to confirm the diagnosis. Contrary to PAM, lumbar puncture with CSF may not show trophozoites due to the encysted nature and brain biopsy may be needed. The CSF shows mild lymphocytic pleocytosis, elevated protein, and low glucose. Trophozoites are rarely seen in CSF because of their encysted nature. However, Giemsa stain could show the trophozoites [10]. In the hematoxylin and eosin (H&E) stain of fixed preparation of CSF, acanthamoeba trophozoites is visualized, which may also indicate trophozoites [9].

Balamuthia mandrillaris (M) causes GAE. It can be isolated from soil, dust, and water [11]. It preferably affects immunocompromised patients but also affects the immunocompetent [4]. Currently, 200 cases have been reported, predominantly in the United States and South America. Hispanic patients appeared to be more affected due to possible genetic susceptibility and environmental exposure. The infection seems to be more common in males [11]. The course toward a fatal outcome since the onset of neurologic symptoms is two to 12 weeks if untreated. The mortality rate is around 95% [4]. The clinical features include neurological and dermatological findings. Patients present with annular, nonulcerated, and infiltrative plaques in the central face over the nose. The main neurological finding is thrombotic angiitis, which leads to infarction, hemorrhage, and necrosis. Other neurological signs included unilateral headaches, regression, focal seizures, and cranial nerve dysfunction. Patients can present with localized motor deficits followed by meningeal signs and elevated intracranial pressure. Finally, patients evolve to a progressive loss of consciousness [4,11]. To diagnose these infections, we must have clinical suspicion based on a history of neurologic findings and dermatological findings. While presentations may be different, overall, they have similar central nervous system (CNS) symptoms. Histopathology of the skin affection reveals a diffuse granulomatous reaction in the reticular dermis, granulomas, an infiltrate rich in plasma cell and lymphocytes, and abundant giant cells inside and outside the granulomas [12-13]. Visualization of a trophozoite is required for definitive diagnosis in 60%-75% of cases. Direct immunofluorescence or immunoperoxidase staining must be performed if possible [13]. Additionally, a new polymerase chain reaction (PCR) technique for the detection of amebic deoxyribonucleic acid (DNA) has been recently introduced [12-13]

Table 1 shows the main epidemiological, clinical, and diagnostic features of free-living amoebas [1-13].

**Table 1:** Clinical features of free-living amoebas CSF: cerebrospinal fluid; ICP: increased intracranial pressure; CNS: central nervous system

	Naegleria	Acanthamoeba	Balamuthia
Epidemiology	Lives on surfaces with rich biofilm. Risk factors: hematological malignancies, diabetes mellitus, prolonged use of antibiotics, immunosuppressive state. Approximately 150 cases have been reported worldwide. There is no sex predominance	Lives in temperatures above 30 degrees. Most common risk factor: participation in recreational water activities, approximately 300 cases have been reported worldwide. Males predominantly infected	Risk factors: immune-compromised Hispanic origin due to genetic susceptibility and environmental exposure. Approximately 109 cases reported worldwide. Male predominance
Median time from exposure to death	Eight days to several months	9.9 days	Weeks to years
Mortality	97%-98%	99%	95%
Clinical features	Headaches, neck stiffness, ataxia, irritability, photophobia, diplopia, hemiparesis, cranial nerve palsies, and increased ICP	Headaches, behavior abnormalities, neck stiffness, ataxia, irritability, photophobia, diplopia, high fever, meningeal signs, cranial nerve palsies, seizures, encephalitis, increased ICP, and keratitis	Headaches, behavior abnormalities, neck stiffness, ataxia, irritability, photophobia, diplopia, high fever, meningeal signs, cranial nerve palsies, seizures, encephalitis, increased ICP, and skin manifestations followed by a neurological compromise in weeks or months. The skin signs are: annular, non-ulcerated, infiltrative, asymptomatic plaque in the central face over the nose
Diagnosis	Microscopic findings reveal granulomas with multinucleated giant cells. Specific antibodies to different species of Acanthamoeba along with immunofluorescent staining can also be used.	CSF examination by the phase-contrast microscope is beneficial for ameba visualization of Giemsa or trichrome stains help to define morphology features	Recognition of the cutaneous and CNS findings. Visualization of the parasite is required for definitive diagnosis and is acquired by biopsy
Imaging	On CT/MRI, both enhancing and non-enhancing lesions can be seen. There are also multifocal areas of signal intensity or discrete lesions that may be seen,	CT scan and MRI findings are nonspecific. However, brain edema, hydrocephalus, basilar meningeal enhancement, and infarctions are described. These lesions are found on the frontal and temporal lobes, cerebellum, and spinal cord	Multiple lesions from small and solid to large and nodular lesions with ring enhancement surrounding vasogenic edema and regional mass effect may be present as intralesional hemorrhage, lesions can compromise both white and gray matter.
CFS Findings	Glucose: Low/Protein: High/CSF pressure: increased, Cells: pleocytosis with abundant lymphocytes and polymorphonuclear leukocytes	Glucose: Low/Protein Hiigh CSF pressure: increased/Cells: pleocytosis with abundant lymphocytes and polymorphonuclear leukocytes. Also red cells	Glucose: Normal to low/Protein: Normal to elevated/CSF pressure: Increased/cells: pleocytosis with abundant lymphocytes and polymorphonuclear leukocytes
Empiric Treatment	Initial dose of amphotericin B (IV) for three days, followed by amphotericin B (IV) with a smaller quantity for 14 days. Then, a regimen of azithromycin (IV/PO), rifampin (IV/PO), miltefosine, (IV/PO and dexamethasone (IV)	Pentamidine (PO), Sulfadiazine (PO), Flucytosine (PO), Fluconazole (PO or IV), miltefosine (PO), and azithromycin (PO). The duration of the treatment has not been established	Optimal treatment is uncertain

As mentioned before, these infections are mostly fatal, with mortality rates ranging from 95%-99%[1,6,11]. These concerning numbers demand that research be done to look for treatments that lower mortality rates. Lately, the drug miltefosine has been shown to reduce the growth of amoeba and eliminate the drug in vitro studies. This paper aims to review the reports where miltefosine has been used to determine if adding miltefosine decreases the fatality rate. During this review, we aim to consolidate the knowledge of miltefosine use in treating these infections. Also, we aim to investigate the efficacy of the drug and develop a comprehensive overview of the latest miltefosine use in treating these infections

## Review

Methods

We used a medical subject strategy (MeSH) in PubMed for the realization of this paper. We included articles published in the last 20 years, in the English Language, and conducted on humans. We excluded literature reviews, systematic reviews, and meta-analyses from the discussion of this publication.

Table 2 shows the combination of MeSH terms used to discuss this article and the number of articles found with each MeSH term combination.

**Table 2:** Methods of the study MeSH: medical subject subheading

Search Term	Papers extracted
("miltefosine" [Supplementary Concept]) AND “Balamuthia"[Mesh]	1
("miltefosine" [Supplementary Concept]) AND "Acanthamoeba"[Mesh]	2
("miltefosine" [Supplementary Concept]) AND "Naegleria fowleri"[Mesh]	9
(miltefosine[Title/Abstract]) AND (naegleria[Title/Abstract])	23
(miltefosine[Title/Abstract]) AND (balamuthia[Title/Abstract])	13
(miltefosine[Title/Abstract]) AND (acanthomoeba[Title/Abstract])	44

Results

We initially screened 102 publications. After applying the inclusion and exclusion criteria, we had 92 papers. We excluded 14 more papers because either the data could not be extracted or the paper was not relevant for our objectives or study outcomes. We exclude papers that were literature reviews, systematic reviews, or meta-analyses and ended up with 52 papers.

Table 3 shows the results of the study.

**Table 3:** Results of the study MeSH: medical subject heading

MeSH term combination	Number of articles
Initial recollection of information	102
Paper published in English	92
Paper published after 2010	78
Exclusion of reviews, systematic reviews, and meta-analyses	52

After applying the inclusion and exclusion criteria, we excluded papers that did not meet our objectives or were duplicates and ended up with 24 papers for the discussion (not including the introduction) of this paper.

Discussion

First, a historical review of the drug miltefosine is given. Then, the drug's role is analyzed in the treatment of the three FLA (Naegleria, Balamuthia, and Acanthoamabe) meningoencephalitis. The main points to review are the in vitro studies, animal studies, and studies conducted on humans.

Milfetosine Historical Review

Miltefosine was implemented in 1980 as an experimental antineoplastic agent for breast cancer [14]. Moreover, in 2002, it was used as an antiparasitic for a case of visceral leishmaniasis [2]. In 2013, the CDC started recommending miltefosine for the treatment of PAM. Later, in 2014, the Food and Drug Administration (FDA) authorized its use in Leishmaniasis's treatment in patients over 12 years of age. Additional therapeutic benefits have been reported in American and African trypanosomiasis [14-15]. The precise mechanism of action is not entirely understood. Still, it is known that the phospholipid structure and alkyl phosphocholine compound allow the drug to penetrate the blood-brain barrier and concentrate in brain tissue [14-15]. Miltefosine is usually well-tolerated, except for gastrointestinal complaints, nephrotoxicity has also been described [16].

Miltefosine Use in Primary Amoebic Meningoencephalitis

Naegleria causes a robust inflammatory response causing lytic necrosis and hemorrhage [17]. The CDC had reported the use of miltefosine in 26 cases by 2013 [16]. The drug of choice for PAM is amphotericin B for its clinical efficacy. Amphotericin has been used in all reported survivors of PAM [14]. However, the drug has high toxicity levels, so there has been an effort to find new drug alternatives [18]. An in-vitro study compared amphotericin B and miltefosine for a month. The drug's minimal inhibitory concentration (MIC) was 0.78 and 0.25 ug/ml for amphotericin and miltefosine, respectively. There were higher survival rates for miltefosine (55%) as compared to amphotericin B (40%) [15].

It is not known how the dose, frequency, and intervals of administration affect successful treatment. However, there is consensus on a maximum amount of 50 mg tablets: 2.5 mg/kg/day. The treatment period varies from case to case [17,19].

Cases Documenting the Use of Miltefosine in Primary Amoebic Meningoencephalitis

Cope and Dunn reported one and two cases, respectively. Between the three cases, there were two survivors and one death where miltefosine was used. One case had a total functional recovery, the other had chronic neurological manifestation, and the third one died of brain death [18,20].

In another report, a man with no comorbidities presented with a two-day history of fever, worsening headache, and generalized weakness. Neurologic focal deficits were absent. The diagnosis of PAM was made within 24 hours. The CDC guideline was implemented on the first day. On day three, he developed diabetes insipidus (DI) and died four days later with cardiac arrest. Muhammad et al. suggest that the mechanism behind DI in patients with PAM needs to be taken into account regarding the treatment approach in these patients [21].

Two cases mimicked flu-like illness on initial presentation and received supportive care followed by discharge. Meningoencephalitis signs and deleterious neurologic complications developed on the second presentation, followed by early death despite implementing aggressive measures and following the CDC guidelines. Stowe et al. suggest that early diagnosis and assessment of host susceptibility factors should be taken into account for adequate treatment strategies [22]. The following case reports (Table 4) describe the application of miltefosine on second-line use [18,20-22].

**Table 4:** Milfetosine use in primary amoebic meningoencephalitis AmB: amphotericin B, Rif: rifampin, Flu: fluconazole, AZM: azithromycin, Dex: dexamethasone, Acy: acyclovir, Van: vancomycin, CFX: ceftriaxone, Deoxy AmB IV: deoxycholate amphotericin B intravenously, ICU: intensive care unit

Reference	Case (age, sex)	Diagnosis from symptoms onset	Initial treatment¹	Miltefosine initiation from diagnosis	Total days of treatment	Outcome	Follow up
[18]	12-year-old, female	two days	AmB, Rif, Flu, AZM, Dex	36 hours	55 days	Survive	Full recovery after three months, with speech and physical therapy
[20]	12-year-old, male	one day	Acy, liposomal AmB, Rif, Flu, Van, CFX , IV Deoxy AmB, Flu, AZM, Rif	31 hours	16 days	Dead	Braindead on hospital day 16
[20]	Eight-year-old, male	five days	AmB, Rif, Flu, AZM, Dex	14 hours from ICU admission	39 days	Survive	Significant neurological deficits
[21]	44-year-old male	four days	IV/IT AmB, Flu, Rif, Dex	Within 24 hours	four days	Dead	Diabetes Insipidus on day three, Cardiac arrest on day four
[22]	4-year-old male	for – five days	LOR, Phos AZM, AmB, Rif, Flu, Dex	24 - 48 hours	two days	Dead	Braindead on hospital day two
[22]	14-year-old male	three – five days	Van, CFX, Flu, AZM, Rif, IV/IT AmB	24 - 92 hours	four days	Dead	Brain herniation, later braindead on hospital day four

All three patients reported received miltefosine as a second-line drug in their treatment regimen. For such, the outcomes evidently vary from survival with full neurologic recovery, survival with neurological deficits, and death. Two of three patients survive with miltefosine use as compared to three out of nine PAM fatal cases without miltefosine (unpublished reports of the CDC) [18]. Miltefosine's survival advantage depends on its accessibility, availability, and time management. Furthermore, successful recovery is associated with early diagnosis and a prompt establishment of a combination of antimicrobials plus measures to control elevated intracranial pressure [20].

The use of miltefosine looks potentially attractive in the battle against PAM. From its feasible oral administration, its ability to readily accumulate on CSF at amebicidal concentrations with low toxicity offers an advantage over other medications' limitations. At any suspicion of PMA, the CDC should be contacted to consult with an FLA expert for diagnosis assistance, sample collection guidance, and recommendations regarding treatment, including the use of miltefosine [16].

Miltefosine Use in Granulomatous Amebic Encephalitis (Acanthamoeba) 

Acanthamoeba causes GAE. In vitro studies explored miltefosine's effect on three strains of Acanthamoeba: sp, lugdunensis, and castellani. Acanthamoeba castellanii had the highest sensitivity, with about 100% eradication of trophozoites at 62.5 mM after 24 hours [23]. In comparison, the other two strains of Acanthamoeba sp and Acanthamoeba lugdunensis showed resistance even at high concentrations [23].

Topical miltefosine was studied on Syrian hamsters to test its efficacy in treating keratitis. Keratitis due to Acanthamoeba is a painful, life-threatening condition that usually affects immunocompromised patients [24]. At the end of the trial, 85% of corneas treated with miltefosine became cured versus 65% of the other treatment (propamidine isethionate plus polyhexanide) [24].

Cases Documenting the Use of Miltefosine in Granulomatous Amebic Encephalitis (Acanthamoeba) 

In a case report, a 60-year-old woman with rhinorrhea and sinus pressure five months after heart transplantation was treated with ampicillin-sulbactam [25]. On MRI, a raised right nasal septal mass was discovered. The mass was resected and sent for histological examination, revealing the diagnosis of GAE [25]. The infection spread to the skin, sinuses, and bones. Initial therapy was amphotericin B and metronidazole for four days [25]. On day four, the regimen switched to a combination of fluconazole, flucytosine, and miltefosine [25]. Six months after treatment, the lesions resolved, and miltefosine was discontinued eight months after discharge [25]. The patient had a complete recovery.

In a second case report, a 25-year-old immunocompromised man from India developed GAE. The patient also had tuberculous meningitis [26]. The patient was treated with a mixture of miltefosine, amikacin, and four more tuberculostatic drugs. Topical miltefosine cured the skin lesions in six weeks, and it was discontinued after two more additional weeks [26]. Intrathecal amikacin and oral miltefosine were stopped after six and eight weeks, respectively [26]. The patient was eventually discharged for recovery of lesions.

In another report, a 38-year-old man presented with tinnitus for three days before developing a generalized seizure [27]. On CT, a right temporal lobe was found. Several months later, serologic studies consistent with Acanthamoeba came back positive [27]. The patient was admitted to the hospital and administered a combination of miltefosine and voriconazole. The patient fully recovered after he underwent surgical debunking of the mass and a combination of voriconazole and miltefosine for three months [27].

A case report of a 35-year-old man with acquired immunodeficiency syndrome (AIDS) (CD4 lymphocyte count of 30 cells/μl) was diagnosed with GAE on biopsy after MRI imaging showed multiple ring-enhancing lesions with hemorrhages [28]. The patient was started on a combination of miltefosine, fluconazole, albendazole, azithromycin, rifampin, and trimethoprim-sulfamethoxazole [28]. Later, he was surgically intervened but eventually died.

A 35-year-old man complained of recent onset of speech disturbance, gait instability, and a sudden onset of left hemiparesis [29]. MR imaging revealed an inhomogeneous contrast-enhancing right cerebellar lesion [29]. A tumor was suspected, and the patient was started on a steroid for two weeks before surgery. The patient’s condition deteriorated. After confirming his diagnosis with a biopsy, he was treated with fluconazole, trimethoprim-sulphamethoxazole, and miltefosine [29]. The latter was discontinued later for gastrointestinal intolerance. After seven months of treatment, the patient was cured. The author argues that miltefosine only plays a marginal role in treating the infection because of the short duration that the drug was given [29].

A 63-year-old man presented to the emergency department with signs of encephalitis. He received a renal transplant six months ago and was immunocompromised with mycophenolate and tacrolimus [30]. Brain MRI showed intracranial masses in the left frontal lobe and left posterior temporal-occipital with edema [30]. The diagnosis was confirmed with a biopsy on day nine. So, he started with a combination of sulfadiazine, fluconazole, flucytosine, azithromycin, and miltefosine for treatment [30]. His condition continued to deteriorate, and he passed away during his fifth-week post-admission.

Table 5 details the cases of miltefosine use and Acanthamoeba [25-31]

**Table 5:** Case report of a patient with meningoencephalitis due to GAE AmB: amphotericin B, TB: tuberculosis, GAE: granulomatous amebic encephalitis

Reference	Case (age, sex)	Diagnosis from symptoms to onset	Initial treatment	Miltefosine Initiation from diagnosis	Total days of treatment	Outcome	Follow-up
[25]	60 years, female	20 days	AmB, metronidazole and surgical resection	Hospital day four	Six months for resolution of lesions	Survived	Full recovery with eight months of therapy to prevent relapse
[26]	25 years, male	Six weeks	TB regimen, surgical excision, and amikacin	12 weeks after tuberculosis s therapy	Six weeks of amikacin, eight weeks of miltefosine	Survived	Two years follow up without relapse
[27]	38 years, male	Months later	Voriconazole, plus surgical excision	Months	Three months	Survived	Information not found
[28]	35, male	Two weeks	Miltefosine, fluconazole, albendazole, azithromycin, rifampin, and TMX	10 days	Four weeks	Died	He was surgically intervened, but, eventually, died
[29]	35, male	Years	HAART, fluconazole, TMX, and miltefosine	Day 13	Seven days because of gastrointestinal intolerance	Survived	After seven months, he eventually recovered from the infection
[30]	63, male	Two weeks	Dex, surgical excision, sulfadiazine, fluconazole, flucytosine, and azithromycin	10 days	Four weeks	Died	Died due to declining kidney function and pancytopenia

In summary, six case reports where miltefosine was used for Acanthoamoeba were included in this review: five of them survived and two of them died. Even the delayed initiation of miltefosine did not seem to impact the outcome of treatment. The only case that succumbed to death was due to coexisting comorbidities. Miltefosine seemed to be the turning point in the treatment of GAE caused by Acanthamoeba.

Miltefosine Use in Granulomatous Amebic Encephalitis (Balamuthia)

In summary, six case reports of Acanthamoeba were included in this review: five survived, and only one died. Even the delayed initiation of miltefosine didn't seem to impact the outcome of treatment. The only case that succumbed to death was due to coexisting comorbidities. Miltefosine seemed to be the turning point in the treatment of GAE caused by Acanthamoeba.

In the in-vitro studies of miltefosine on Balamuthia, concentrations over 40 mM cause protozoal cell lysis [15]. The mechanism by which the drug killed the parasites was through to be apoptosis [15]. Nonetheless, other reports described that miltefosine also has amoebastatic activity against Balamuthia. In that report, miltefosine inhibited in vitro growth by 90% [15]. Another in vitro study focused on the efficacy of various drugs against FLA, including an analysis of miltefosine against Balamuthia. In this study, other drugs were less effective as compared to miltefosine [32].

The combination of miltefosine, fluconazole, and albendazole is used in most successful cases of Balamuthia's treatment cases. In these cases, it's difficult to determine whether the beneficial outcomes were due to individual or synergistic effects of the drugs used in combinations, which warrants the need for more in vitro sensitivity studies for the medications in question.

Diminazene aceturate, a drug used in African human Trypanosomiasis, was the most effective in vitro. Nevertheless, the drug has significant side effects like polyneuritis, which limits the use in humans [32].

Cases Documenting the Use of Miltefosine in Granulomatous Amebic Encephalitis (Balamuthia)

A 21-year-old woman had four years of cutaneous lesions consistent with cutaneous Balamuthia before hospital admission. The patient received many combinations of treatments that included: fluconazole, clotrimazole, and topical steroids for one year, followed by trimethoprim-sulfamethoxazole, fluconazole, and clarithromycin for eight months [33]. After several more regimens, the patient received a combination of fluconazole, albendazole, and miltefosine. After one year of treatment, magnetic resonance imaging confirmed the disappearance of the intracranial mass [33].

In another case, a 26-year-old Hispanic male presented to the emergency department with symptoms consistent with GAE. He was first treated with high-dose dexamethasone and albendazole for suspicion of neurocysticercosis [34]. He then underwent a brain biopsy and was diagnosed with GAE caused by Balamuthia on the third week of treatment miltefosine. The patient completed 114 combination therapy, including miltefosine, trimethoprim-sulfamethoxazole, azithromycin, and fluconazole [34]. The patient's discharge was approved after the resolution of lesions on MRI [34].

In a case report, a 69-year-old woman was admitted to the hospital for a decreased level of consciousness that started three days before. The woman was HIV negative, but her total CD4+ was 250/mm^3^ [35]. Neuroimaging showed a centrally cavitary lesion in the right frontal lobe, left basal ganglia, and left cerebellar hemisphere [35]. She received IV dexamethasone and underwent craniotomy. The biopsy confirmed GAE by Balamuthia. She received a six medication-regimen of pentamidine, sulfadiazine, azithromycin, fluconazole, flucytosine, and miltefosine [35]. On the third hospital day, she had an ischemic stroke and deteriorated and was moved to hospice care, where she died a few weeks later [35].

Finally, in another case report, an 11-year-old boy of Hispanic ethnicity was hospitalized after three weeks of nausea, vomiting, lethargy, and right-sided weakness that caused him to fall one day before he was admitted to the hospital [36]. The patient was suffering from neurological symptoms for the past few months as well. The patient was sent for an MRI on day one of admission, and a tumor was suspected. On biopsy and H&E stain, trophozoites were present [36]. Therefore, he was diagnosed with GAE, and treatment was started on day three [36]. The patient was placed on miltefosine on day seven, which is when he started showing improvements. However, by day 20, he started having new lesions and his condition began to deteriorate [36]. Miltefosine was stopped on day 34. On day 61, he was pronounced dead [36].

Table 5 details the cases of miltefosine use and Balamuthia [33-36].

**Table 6:** Use of miltefosine for Balamuthia GAE AmB: amphotericin B, Rif: rifampin, Flu: fluconazole, AZM: azithromycin, Dex: dexamethasone, Acy: acyclovir, Van: vancomycin, CFX: ceftriaxone, Deoxy AmB IV: deoxycholate amphotericin B intravenously, TMP-SMX: trimethoprim-sulfamethoxazole, 5-FC: flucytosine, GAE: granulomatous amebic encephalitis

Reference	Case (age, sex)	Diagnosis from symptom to onset	Initial treatment	Miltefosine initiation from diagnosis	Total days of treatment	Outcome	Follow-up
[33]	21, female	Four months	Fluconazole, clotrimazole, topical steroid, fluconazole and clarithromycin, TMP-SMX, albendazole	> two years	one year	Survived	30 months of treatment including 12 months without therapy or recurrence
[34]	26, male	Two months	Dex, albendazole, Azm, Flu,TMP-SMX	Third week	114 days	Survived	Continued nine months of treatment minus miltefosine
[35]	69, female	Three days	Azm, Flu, 5-FC, Sulfadiazine, pentamidine	On 3rd day	Three days	Died	A few weeks after transfer to hospice care
[36]	11, male	Weeks	Dex, AmB, Flu, Azithromycin, 5-FC, Sulfadiazine, pentamidine, metronidazole	On 7th day	27 days	Died	Palliative care on day 49

Overall, four case reports were included in this review on GAE caused by Balamuthia. Miltefosine seemed to be the least effective on GAE caused by Balamuthia in comparison to GAE caused by both N. fowleri and Acanthamoeba. Two cases died despite early treatment with miltefosine due to unclear reasons. In contrast, the two cases that survived received miltefosine relatively late. Miltefosine seems to have a beneficial effect on Balamuthia but not as strong as seen in GAE caused by the other two FLA mentioned in this review.

## Conclusions

Encephalitis caused by free-living amoebas is one of the rarest, yet deadliest, infections that still agitates the medical community in the 21st century. Until today, there are no definitive guidelines to be followed when treating such patients. However, miltefosine has demonstrated promising results. Miltefosine decreases the usual mortality rate in the three infections; however, only a few reports exist due to the low frequency of the infections. The documented cases in our report seem to point out a decrease in the mortality rate as compared to patients treated with standard management. Nonetheless, more research and clinical trials should help set definitive guidelines that can be used worldwide when presented with these life-threatening infections.
